# Molecular epidemiology and pharmacokinetic–pharmacodynamic analysis of *Neisseria gonorrhoeae* strains in Northern Spain

**DOI:** 10.1007/s10096-026-05591-5

**Published:** 2026-07-09

**Authors:** María del Rosario Almela-Ferrer, María Carmen Nieto-Toboso, Elisabeth Montoya-Azpeitia, Josefina López-de Munain, Leonora Hernández-Ragpa, Inigo Gorostiza, Alicia Rodríguez-Gascón, Mikel Urrutikoetxea-Gutiérrez, Andrés Canut-Blasco, Sofía Ibarra-Ugarte, José Luis Díaz de Tuesta-del Arco, Ramón Cisterna-Cancer, Miren Basaras-Ibarzabal

**Affiliations:** 1 Department of Clinical Microbiology, Basurto University Hospital, Osakidetza Basque Health Service, Bilbao, Spain; 2 Microbiology Infection Control Group, Biobizkaia Health Research Institute, Barakaldo, Bizkaia Spain; 3 Department of Infectious Diseases, Basurto University Hospital, Osakidetza Basque Health Service, Bilbao, Spain; 4 Clinical Research in Infectious Diseases Group, Biobizkaia Health Research Institute, Barakaldo, Bizkaia Spain; 5 Research Unit, Basurto University Hospital, Osakidetza Basque Health Service, Bilbao, Spain; 6 Pharmacokinetic, Nanotechnology and Gene Therapy Group (PharmaNanoGene), Faculty of Pharmacy, University of the Basque Country, Vitoria-Gasteiz, Spain; 7 Infectious Disease, Antimicrobial Agents, and Gene Therapy Group, Bioaraba Health Research Institute, Vitoria-Gasteiz, Spain; 8 Department of Clinical Microbiology, Araba University Hospital, Osakidetza Basque Health Service, Vitoria-Gasteiz, Spain; 9 Department of Immunology, Microbiology and Parasitology, University of the Basque Country, Leioa, Spain

**Keywords:** *Neisseria gonorrhoeae*, Gonorrhoea, Antimicrobial resistance, NG-MAST, Ceftriaxone, Pharmacodynamics

## Abstract

**Purpose:**

To analyse the epidemiology, antimicrobial susceptibility and molecular diversity of *Neisseria gonorrhoeae* isolates recovered in 2016–2017 at a tertiary hospital in northern Spain, and to perform a seven-year pharmacokinetic/pharmacodynamic (PK/PD) evaluation using susceptibility data from previous studies conducted in the same hospital setting.

**Methods:**

A total of 231 non-duplicated isolates from 227 patients were studied. Antimicrobial susceptibility was determined by MIC gradient strip tests and molecular typing was performed by NG-MAST v2.0. Epidemiological variables were analysed, and considering the MIC distribution of the isolates, the cumulative fraction of response (CFR) for relevant antimicrobials was estimated.

**Results:**

Most infections occurred in men (80.5%), particularly among men who have sex with men (50.3%), with the highest incidence in patients aged 25–34 years. All isolates were susceptible to ceftriaxone and cefixime (100%), while susceptibility to azithromycin remained above 95% throughout the study period. In contrast, ciprofloxacin resistance remained high, ranging from 46.2% to 72.7% over the seven-year period. PK/PD analysis showed that CFR values ≥ 90% were achieved only with cephalosporins. Molecular typing identified 91 STs, including 15 previously undescribed STs. Six genogroups accounted for 51.9% of isolates and were associated with distinct epidemiological and resistance patterns.

**Conclusion:**

Ceftriaxone remains the preferred empirical treatment option for gonococcal infection. The favourable PK/PD performance observed for cefixime supports its activity against the studied isolates, although current treatment recommendations continue to prioritize ceftriaxone, particularly for extragenital infections; whereas ciprofloxacin should not be recommended for empirical use because of persistently high resistance rates. Continued molecular and antimicrobial resistance surveillance is essential for monitoring the emergence and dissemination of NG-MAST genogroups associated with antimicrobial resistance and for guiding future treatment strategies.

**Supplementary Information:**

The online version contains supplementary material available at https://doi.org/10.1007/s10096-026-05591-5.

## Introduction

 Gonococcal infection is the second most common bacterial sexually transmitted infection (STI) after *Chlamydia trachomatis* and represents a major public health concern due to its high prevalence and potential complications. According to the World Health Organization (WHO), approximately 82.4 million new cases of gonococcal infection were estimated worldwide in 2020 [1, 2].

Current guidelines, including those from the WHO [3] and the British Association for Sexual Health and HIV [4], recommend ceftriaxone monotherapy as first-line treatment. However, the increasing emergence of antimicrobial resistance in *Neisseria gonorrhoeae* (NG) has significantly compromised the effectiveness of previously recommended therapies [5–7]. In 2015, the FC428 clone associated with ceftriaxone resistance was first identified in Japan [8–11] and has since spread internationally [12]. In Europe, the European Gonococcal Antimicrobial Surveillance Programme (Euro-GASP), coordinated by the European Centre for Disease Prevention and Control, provides essential data for monitoring resistance and informing treatment guidelines [13]. However, local epidemiological and resistance patterns remain essential to guide empirical therapy at the regional level.

Pharmacokinetic/pharmacodynamic (PK/PD) analysis is a key tool for optimizing antimicrobial therapy. Combined with Monte Carlo simulation, it allows optimization of treatment regimens in order to maximize efficacy, minimize adverse effects and reduce the emergence of resistance [14]. This approach integrates drug exposure at the site of infection with bacterial susceptibility, expressed as the minimum inhibitory concentration (MIC) [15]. In a recent study conducted in Spain, PK/PD analysis demonstrated that cephalosporins provide a high probability of treatment success, supporting their role as first-line agents for gonococcal infection [16].

Molecular epidemiological surveillance is essential to understand the distribution of NG genogroups, their association with antimicrobial resistance patterns, and the emergence and spread of resistant clones.

The aim of this study was to analyse the epidemiology, antimicrobial susceptibility and molecular characteristics of gonococcal strains isolated between 2016 and 2017 in a tertiary hospital in northern Spain, and to perform a 7-year PK/PD evaluation using susceptibility data from previous studies conducted in the same hospital setting [17].

## Materials and methods

This was a retrospective observational study conducted at the Clinical Microbiology Service of Basurto University Hospital, a tertiary teaching hospital in Bilbao serving a population of 356,000 inhabitants. The study period covered 2016–2017. This hospital provides specialist care for sexually transmitted infections (STIs) and implements active partner management and contact tracing.

For the estimation of gonococcal infection incidence, both culture-positive cases and those diagnosed exclusively by molecular methods were considered. Primary molecular diagnosis was performed using the BD MAX™ System (Becton Dickinson, USA) with the BD CTGCTV2 assay. However, all subsequent analyses—including antimicrobial susceptibility testing, molecular typing and PK/PD analyses—were restricted to culture-confirmed isolates to ensure consistency in microbiological procedures requiring viable organisms. Accordingly, all non-duplicated *Neisseria gonorrhoeae* (NG) isolates recovered by culture were included in the microbiological analyses.

Gonococcal isolation was performed on chocolate agar and GC-LECT agar (Becton Dickinson, USA). Presumptive colonies were identified by MALDI-TOF MS (Microflex LT, Bruker-Daltonics, Bremen, Germany).

The study was approved by the Clinical Research Ethics Committee of Basurto University Hospital (internal code: 48.18CEICHUB).

### Study design and unit of analysis

The primary unit of analysis was the episode of infection, defined by each isolate obtained. Recurrent infections in the same patient were considered independent episodes when clinical cure had been documented following a previous episode. Although certain variables are presented at patient level for descriptive purposes, the episode was consistently considered the unit of analysis throughout the study.

### Epidemiological survey

Incidence rates were calculated per 100,000 inhabitants using the reference population served by the hospital, including both culture-confirmed cases and those diagnosed exclusively by molecular methods. Socio-demographic and clinical data were obtained from the clinical records. The variables analysed were age, sex, reason for consultation, geographical origin, sexual behaviour, site of infection, human immunodeficiency virus (HIV) status, co-infection with other STIs, history of previous STIs, involvement in prostitution or being a prostitution client, and number of sexual contacts during the previous 12 months.

### Antimicrobial susceptibility

Antimicrobial susceptibility was determined using MIC gradient test strip assays (Liofilchem, Roseto degli Abruzzi, Italy) on GC agar base (Difco GC Medium Base, Becton Dickinson) supplemented with 1% Vitox (Oxoid) according to CLSI recommendations [18]. The MICs of penicillin, cefixime, ceftriaxone, azithromycin, ciprofloxacin, tetracycline, spectinomycin and gentamicin were determined. Resistance rates were calculated according to the European Committee on Antimicrobial Susceptibility Testing (EUCAST) [19]. For gentamicin, susceptibility was defined as MIC ≤ 4 mg/L, intermediate susceptibility as MIC between 8 and 16 mg/L and resistance as MIC ≥ 32 mg/L according to Poncin et al. [2].

### Pharmacokinetic/pharmacodynamic (PK/PD) analysis

Considering the published pharmacokinetic parameters and the PK/PD targets shown in Table 1 [20–24] and the MIC distribution of the isolates, we estimated the probability of treatment success for the antibiotics by calculating the cumulative fraction of response (CFR), defined as the expected population probability of target attainment (PTA) for a given dose against the bacterial population. The PTA represents the probability that the PK/PD index reaches the predefined target. The following equation was used to calculate the CFR:

$$\:CFR\left(\%\right)={\sum\:}_{i=1}^{n}{PTA}_{i}\times\:\:{F}_{i}$$

where i indicates the MIC category, PTA_i_ is the PTA for each MIC category, and F_i_ is the fraction of the microorganism population within each MIC category.

**Table 1:** Pharmacokinetic/pharmacodynamic targets, dosing regimens and pharmacokinetic parameters used for Monte Carlo simulations

Antimicrobial agent	Dosage regimen evaluated	Route	PK/PD index	Target value	Pharmacokinetic parameters used	Reference(s)
Ciprofloxacin	500 mg SD	Oral	AUC_0_–_∞_/MIC	≥ 125	AUC₀–∞ = 10.7 ± 2.6 mg·h/L	[20, 21]
Azithromycin	1 g SD; 2 g SD	Oral	AUC_0_–_∞_/MIC	≥ 59.5	CL/F = 144 ± 39.5 L/h; Ke = 0.082 ± 0.029 h⁻¹	[22, 23]
Ceftriaxone	250 mg SD; 500 mg SD; 1 g SD	IM	fT > MIC	≥ 20 h	Vd = 14.70 ± 4.93 L; Fu = 0.05	[24]
Cefixime	400 mg/day	Oral	fT > MIC	≥ 60% of dosing interval	F = 0.42 ± 0.045; Ka = 1 h⁻¹; Ke = 0.204 ± 0.02 h⁻¹; Vd = 19 ± 0.03 L; Fu = 0.35	[24]

*PK/PD* pharmacokinetic/pharmacodynamic, *SD* single dose, *AUC₀–∞* area under the concentration–time curve from zero to infinity;, *MIC* minimum inhibitory concentration,; *CL/F* apparent oral clearance, *Ke* elimination rate constant, *IM* intramuscular, *fT > MIC* percentage of time during which free drug concentrations remain above the MIC, *Vd* volume of distribution, *Fu* unbound fraction, *F* bioavailability, *Ka* absorption rate constant

Adapted from Alonso et al. [16] under the terms of the Creative Commons CC BY 4.0 license

For each antibiotic and MIC value, we used the PTA values reported in a previous study [16]. For empirical treatment, a dosing regimen was considered effective when it achieves CFR ≥ 90%, whereas CFR values ≥ 80% but < 90% were interpreted as indicative of moderate efficacy.

### Molecular typing

NG strains were typed using the *Neisseria gonorrhoeae* multi-antigen sequence typing (NG-MAST v2.0) method, as described by Martin et al. [25]. This technique differentiates the strains according to sequence variations in internal fragments of two genes: *porB* (encoding the PorB porin) and *tbpB* (encoding transferrin-binding protein subunit B). The PubMLST database (https://pubmlst.org/organisms/neisseria-spp) was used to assign the alleles and sequence types (ST). Isolates without an ST match were uploaded to PubMLST for assignment of new STs. In addition, the alleles from the predominant STs (represented by ≥ 10 isolates) were classified into genogroups as described by Chisholm et al. [26]. Genogroups were designated using the letter “G” followed by the predominant ST of each group.

### Statistical analysis

Continuous variables were described as mean and standard deviation (SD) when they met the criteria for a normal distribution, assessed using the Kolmogorov-Smirnov test. Frequencies and percentages were used to describe qualitative variables. Differences between groups were assessed using Fisher’s exact test for categorical variables. Student’s t-test was used to compare means of quantitative variables. Although certain variables are presented at patient level for descriptive purposes, the episode was consistently considered the unit of analysis throughout the statistical analysis. Missing data were present in some epidemiological variables; consequently, denominators vary across analyses and percentages were calculated based on the available data for each variable. A *p*-value < 0.05 was considered statistically significant.

CFRs were calculated over a 7-year period. Temporal trends in antimicrobial susceptibility and CFR values were assessed using linear regression analysis, with calendar year as the independent variable. For each antimicrobial agent and regimen, the annual percentage values were modelled to estimate the slope (β coefficient), coefficient of determination (R²), and statistical significance (*p*-value). A *p*-value < 0.05 was considered indicative of a statistically significant trend. For variables with no variability over time, defined as values remaining constant at 100% throughout the study period, regression analysis was not performed.

Data were analysed using Stata v18.

## Results

### Epidemiological survey

The incidence of gonococcal infection was 50 cases per 100,000 inhabitants in 2016 (178 detections) and 48 cases per 100,000 inhabitants in 2017 (171 detections), with no significant variation observed between the two years. A total of 231 NG isolates (231 episodes of infection) were recovered from 227 patients. Four patients experienced two episodes of gonococcal infection. A total of 186 episodes (80.5%) occurred in men and 45 (19.5%) in women. The mean age was 31 years (SD = 9.6, range 18–69 years). The most affected age group was 25–34 years, with 94 cases (40.7%). Statistically significant differences were found between age groups and sex (*p* = 0.028), with women being younger than men. Regarding nationality, of the 231 episodes, 179 (77.5%) occurred in patients from Spain.

Sexual behaviour data were available for 191 episodes: 96 (50.3%) corresponded to men who have sex with men (MSM), 50 (26.2%) to men who have sex with women (MSW) and 45 (23.6%) to women (all reporting sex with men).

Reason for consultation was known for 159 episodes, including 126 in men and 33 in women. Most consultations were due to clinical manifestations (*n* = 105; 66.6%), followed by risky sexual behaviour (*n* = 34; 21.4%) and screening programs (*n* = 20; 12.6%). Among the 126 men with available data, 94 (74.6%) consulted because of clinical symptoms, whereas among the 33 women with available data, 18 (54.5%) were diagnosed after being identified as close contacts of an infected person. The reason for consultation showed a statistically significant association with sex (*p* < 0.001).

A history of previous STIs was identified in 76 of 152 episodes (50%) and was significantly associated with sexual behaviour (*p* = 0.005), being more frequent among MSM (49/78; 62.8%).

Of the 231 episodes, most isolates were obtained from urethral samples (*n* = 140; 60.6%), followed by rectal (*n* = 78; 33.8%), pharyngeal (*n* = 71; 30.7%) and endocervical (*n* = 39; 16.9%).

HIV status was available for 177 patients (corresponding to 177 episodes), of whom 19 (10.7%) were positive. In 58/191 episodes (30.4%) patients had at least one concurrent STI other than HIV, showing a significant association with sexual behaviour (*p* = 0.031): the prevalence was higher in women (*n* = 16; 35.6%) and MSM (*n* = 34; 35.4%) than in MSW (*n* = 8; 16%). Coinfection with *Chlamydia trachomatis* was the most frequent concurrent STI, accounting for 46 of the 58 coinfections (79.3%).

Contact with prostitution was reported in 11 patients/191 (5.8%), all of whom were MSW. The mean number of sexual contacts during the previous 12 months was 10 (SD = 18.7).

### Antimicrobial susceptibility

**Fig. 1:**
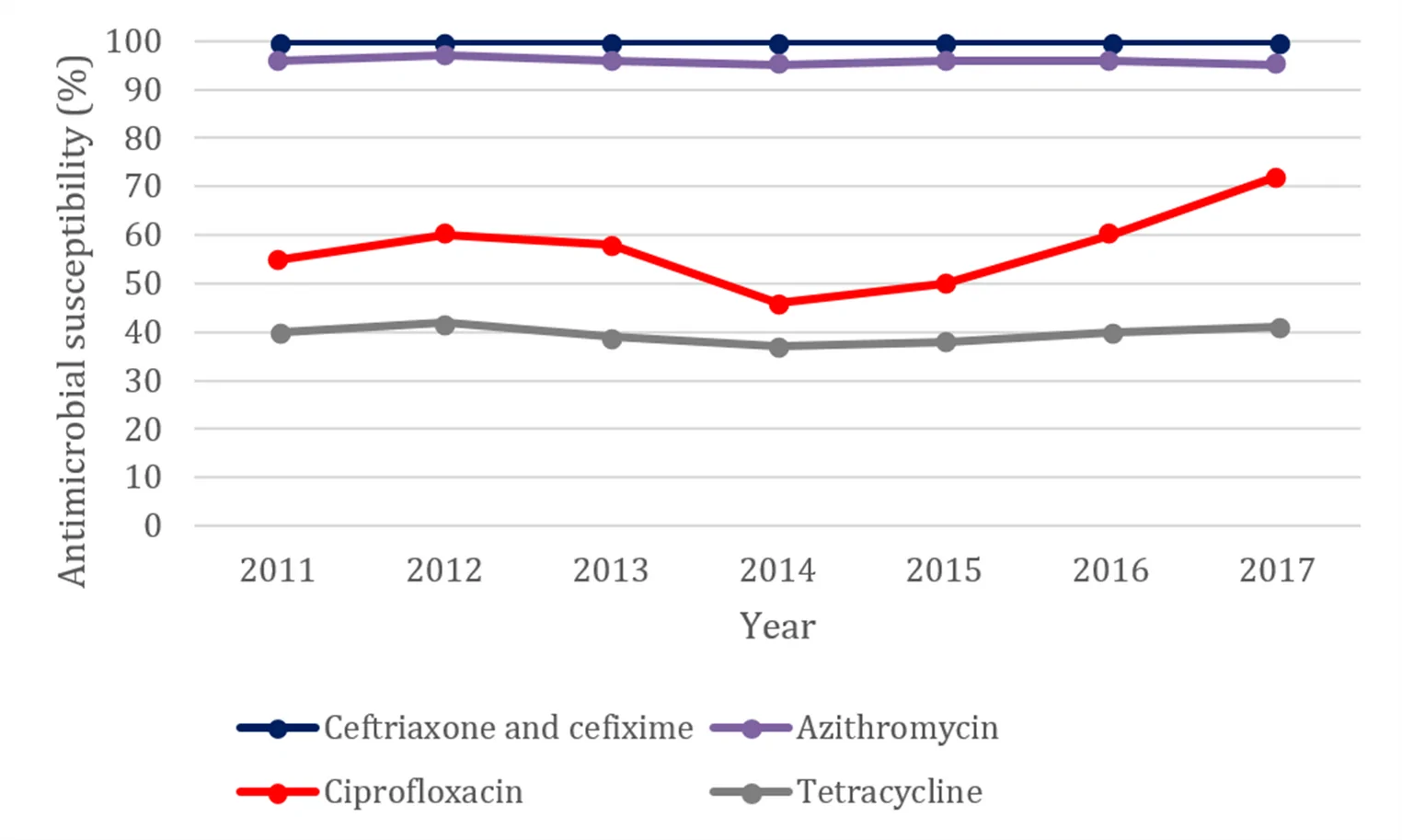
Temporal trends in antimicrobial susceptibility of *Neisseria gonorrhoeae* isolates, 2011–2017. Susceptibility rates are expressed as the percentage of susceptible isolates for each antimicrobial agent

When considering the extended study period (2011–2017), susceptibility to azithromycin remained stable throughout the study period, consistently exceeding 95%. Ciprofloxacin susceptibility showed greater variability, with a decrease in 2014 followed by a subsequent increase in later years. Temporal trends revealed no statistically significant changes in antimicrobial susceptibility over time. Susceptibility to cefixime and ceftriaxone remained constant at 100% throughout the study period, precluding regression analysis for these agents. Detailed trends are presented in Table 2 and Fig. 1.

**Table 2:** Annual susceptibility rates of *Neisseria gonorrhoeae* isolates to selected antimicrobials during 2011–2017

Antimicrobial agent	2011 (%)	2012 (%)	2013 (%)	2014 (%)	2015 (%)	2016 (%)	2017 (%)	Slope (% per year)	*R*^2^	*p*-value		Trend
Azithromycin	97.4	96.0	96.8	98.4	98.2	97.3	95.8	−0.03	0.004	0.896	No significant	
Ciprofloxacin	33.1	23.5	34.5	18.0	51.4	41.8	45.2	+ 3.21	0.341	0.168	No significant	
Cefixime	100	100	100	100	100	100	100	0	-	-	Constant	
Ceftriaxone	100	100	100	100	100	100	100	0	-	-	Constant	

### PK/PD analysis

**Fig. 2:**
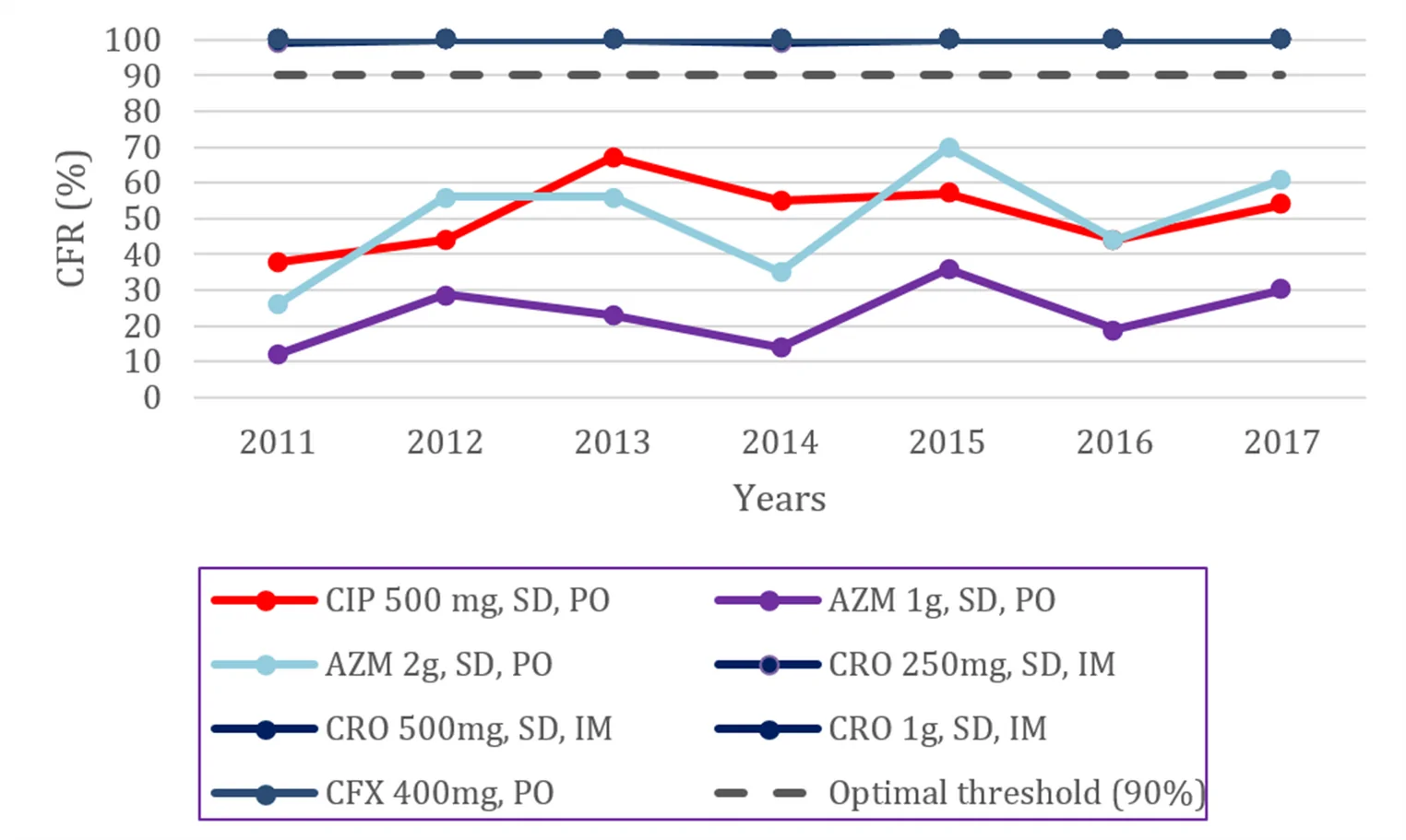
Temporal trends in cumulative fraction of response (CFR) for selected antimicrobial regimens against *Neisseria gonorrhoeae*, 2011–2017. *Abbreviations*: CIP, ciprofloxacin; AZM, azithromycin; CRO, ceftriaxone; CFX, cefixime

Table 3 and Fig. 2 summarize the CFR values obtained for the antibiotics included in the study, and their temporal trends. CFR values ≥90% were achieved exclusively with cephalosporins. For ceftriaxone 500 mg, ceftriaxone 1 g and cefixime 400 mg/day, CFR values remained constant at 100% throughout the study period, precluding regression analysis for these regimens.

**Table 3:** Annual cumulative fraction of response (CFR) values according to antimicrobial regimen during 2011–2017

Antimicrobial	Dose	2011	2012	2013	2014	2015	2016	2017	Slope^a^	*R* ^2^	*p*-value	Trend
Ciprofloxacin	500 mg, SD, PO	38	44	67	55	57	44	54	+ 1.36	0.088	0.517	No significant
Azithromycin	1 g, SD, PO	12	29	23	14	36	19	30	+ 1.68	0.167	0.362	No significant
	2 g, SD, PO	26	56	56	35	70	44	61	+ 3.39	0.225	0.282	No significant
Ceftriaxone	250 mg, SD, IM	99	100	100	99	100	100	100	+ 0.11	0.225	0.282	No significant
	500 mg, SD, IM	100	100	100	100	100	100	100	0	-	-	Constant
	1 g, SD, IM	100	100	100	100	100	100	100	0	-	-	Constant
Cefixime	400 mg/day, PO	100	100	100	100	100	100	100	0	-	-	Constant

Values represent CFR (%). ^a^ Percentage per year

*SD* single dose, *PO* oral, *IM* intramuscular

### Molecular characterization

Molecular characterization was performed on 231 strains: nineteen strains had no match in the database, whereas the remaining isolates were assigned to 91 different STs, comprising 69 *porB* alleles and 31 *tbpB* alleles. The most prevalent STs were ST5441 (*n* = 25; 10.8%), ST51 (*n* = 19; 8.2%), ST9184 (*n* = 14; 6.1%) and ST13489 (*n* = 13; 5.6%). All detected STs and their corresponding alleles are shown in Supplementary Table S1 (Supplementary material). Isolates without a match to a known ST were submitted to PubMLST and assigned a new ST. These newly assigned STs are shown in Table 4.

**Table 4:** Newly identified NG-MAST sequence types and associated demographic and antimicrobial susceptibility characteristics

ST assigned (*n*)	NG-MAST porB	NG-MAST tbpB	Age	Sex	Sexual behaviour	Azithromycin MIC (mg/L)	Cefixime MIC (mg/L)	Ceftriaxone MIC (mg/L)	Ciprofloxacin MIC (mg/L)	Penicillin MIC (mg/L)	Spectinomycin MIC (mg/L)	Tetracycline MIC (mg/L)
ST25132 (1)	90	2810	26	F	WSM	0.25	< 0.016	0.002	2	0.25	8	0.25
ST25133 (2)	1142	897	31	M	MSM	0.125	< 0.016	0.008	2	0.064	4	0.25
ST25134 (2)	1700	953	37	F	WSM	0.25	0.016	0.016	1	0.5	8	0.25
ST25135 (1)	5942	188	42	M	MSW	0.25	< 0.016	0.008	< 0.002	0.064	8	0.25
ST25136 (1)	372	29	28	F	WSM	0.25	< 0.016	0.004	< 0.002	0.125	8	0.25
ST25137 (1)	1142	263	47	M	MSM	0.25	0.016	0.002	0.004	0.125	8	0.5
ST25138 (1)	1142	50	25	M	MSW	0.125	< 0.016	0.002	0.002	0.125	8	0.5
ST25139 (1)	5475	29	26	F	WSM	0.5	< 0.016	0.004	0.004	0.125	8	0.5
ST25140 (1)	372	563	21	M	MSM	0.125	0.016	0.016	2	0.25	8	0.5
ST25141 (1)	39	563	25	M	MSW	0.5	0.016	0.032	8	0.25	8	0.5
ST25142 (2)	124	50	29	F	WSM	0.25	< 0.016	0.004	0.002	0.125	16	0.5
ST25143 (1)	11,460	4	32	M	MSM	0.25	0.032	0.008	0.25	0.5	4	1
ST25144 (1)	28	121	24	F	WSM	0.125	< 0.016	0.008	0.5	2	4	8
ST25145 (2)	4351	60	25	M	MSM	0.125	< 0.016	0.002	2	1	8	8
ST25146 (1)	2193	1035	42	M	MSW	0.064	< 0.016	0.004	< 0.002	0.125	4	16

*F* female, *WSM* women who have sex with men, *M* male, *MSM* men who have sex with men, *MSW* men who have sex with women

Six genogroups were defined, encompassing 120 isolates (51.9% of the total). To ensure consistency with the current PubMLST NG-MAST v2.0 nomenclature, sequence types ST5119, ST4198 and ST4684, which were present in the database when the molecular analyses were performed, were reassigned to the currently valid genogroup designation G2992, following database updates implemented in 2022 (See Table 5).

**Table 5:** Major NG-MAST genogroups identified among *Neisseria gonorrhoeae* isolates based on *porB* and *tbpB* allele similarity

Genogroup *n* (%)	Predominant ST (*n*)	ST	
		Same porB and ≥ 99% tbpB (*n*)	Same tbpB and ≥ 99% porB (*n*)
G5441 44 (19)	**ST5441** (25)	-	**ST13489** (13), **ST14299** (2), **ST18362** (2), **ST12711** (1), **ST15923** (1)
G9184 20 (8.7)	**ST9184** (14)	-	**ST2400** (3), **ST10128** (2), **ST15707** (1)
G51 20 (8.7)	**ST51** (19)	-	**ST239** (1)
G16377 16 (6.9)	**ST16377** (5)	-	**ST5619** (4), **ST13863** (2), **ST5622** (2), **ST12394** (1), **ST13123**(1), **ST17407** (1)
G11461 10 (4.3)	**ST11461** (7)	-	**ST15724** (2), **ST14764** (1)
G2992 10 (4.3)	**ST5119**^**a**^ (4)	-	**ST4198**^**a**^ (2), **ST2992** (3), **ST4684**^**a**^ (1)

^a^ ST5119, ST4198 and ST4684 were valid sequence types in the NG-MAST database at the time of analysis but were removed following database updates implemented in 2022

G5441 (*n* = 44; 19%) was the largest genogroup and included the highest number of STs. G51 and G9184 were the second largest genogroups with 20 isolates each (8.7%). Associations between genogroups and epidemiological characteristics are shown in Table 6.

**Table 6:** Epidemiological characteristics associated with the predominant NG-MAST genogroups

	G5441 (44)	G9184 (20)	G51 (20)	G16377 (16)	G11461 (10)	G2992 (10)	Total 120 (100)	*p*
Mean age, years (range)	32 (18–55)	32 (21–56)	24 (18–40)	30 (21–61)	35 (23–47)	32 (23–44)		
Spanish (%)	33 (75)	19 (95)	11 (55)	11 (68.8)	10 (100)	9 (90)	93 (77.5)	0.014
M (%)	40 (90.9)	16 (80)	11 (55)	10 (62.5)	10 (100)	8 (80)	95 (79.2)	0.005
Known sexual behaviour, n	34	17	15	14	6	9	95	
MSM (%)	22 (64.7)	3 (17.6)	2 (13.3)	2 (14.3)	6 (100)	6 (66.7)	41 (43.2)^a^	0.001
HTX (%)	12 (35.3)	14 (82.4)	13 (86.7)	12 (85.7)	0 (0)	3 (33.3)	54 (56.8)^a^	0.001
Other STIs (%)	11 (25)	6 (30)	12 (60)	3 (18.8)	3 (30)	1 (10)	36 (30)	0.047
HIV (%)	4 (9.5)	1 (5.3)	0 (0)	1 (7.1)	1 (11.1)	0 (0)	7 (36.8)^b^	0.784
Contact with prostitution	3 (6.8)	4 (20)	0 (0)	2 (12.5)	0 (0)	0 (0)	9 (7.5)	0.184
Number of patients with known number of sexual contacts	44	20	20	16	10	10	120	
≥ 2 sexual contacts (%)	25 (56.8)	7 (35)	12 (60)	11 (68.8)	4 (40)	7 (70)	66 (55)	0.274

^a^From number of patients with known sexual behaviour within genogroups. ^b^ From total of HIV positive patients

*M* man, *MSM* men who have sex with men, *HTX* heterosexual patients, *HIV* human immunodeficiency virus

All genogroups were more frequent among Spanish patients and men, except for G51, which was similarly distributed between Spanish and foreign patients and between men and women (*p* < 0.05). A statistically significant association was found between sexual behavior and genogroups, with heterosexual men and women being more frequently affected by G51, G9184 and G16377, whereas G11461 was detected exclusively in MSM (*p* < 0.001).

The relationship between genogroups and antimicrobial resistance is shown in Table 7.

**Table 7:** Antimicrobial susceptibility profiles according to predominant NG-MAST genogroups

	G5441 (44)	G9184 (20)	G51 (20)	G16377 (16)	G11461 (10)	G2992 (10)	*p*
β-lactamase positive	1 (2.3%)	0 (0%)	0 (0%)	0 (0%)	0 (0%)	0 (0%)	-
CRO modal MIC (range)	0.004 (< 0.002–0.008)	0.016 (0.002–0.064)	0.004 (< 0.002–0.008)	0.032 (0.008–0.064)	0.004 (< 0.002–0.008)	0.004 (0.002–0.008)	-
CFX modal MIC (range)	< 0.016 (< 0.016)	0.032 (< 0.016–0.064)	< 0.016 (< 0.016)	0.064 (< 0.016–0.125)	< 0.016 (< 0.016)	< 0.016 (< 0.016)	-
CN modal MIC (range)	4 (2–8)	4 (0.5–4)	4 (2–8)	4 (4–8)	8 (4–8)	8 (4–8)	-
Penicillin R (%)	0 (0)	0 (0)	1 (5)	2 (12.5)	0 (0)	0 (0)	0.369
Tetracycline R (%)	0 (0)	7 (35)	0 (0)	12 (75)	7 (70)	0 (0)	< 0.001
Azithromycin R (%)	0 (0)	0 (0)	0 (0)	0 (0)	0 (0)	0 (0)	-
Ciprofloxacin R (%)	0 (0)	20 (100)	14 (70)	16 (100)	0 (0)	0 (0)	< 0.001
Spectinomycin R (%)	0 (0)	0 (0)	0 (0)	0 (0)	0 (0)	0 (0)	-

*CRO* ceftriaxone, *CFX* cefixime, *CN* gentamicin, *R* resistant

A statistically significant association was found between ciprofloxacin and tetracycline resistance and genogroups (*p* < 0.001). Genogroups G9184 and G16377 showed 100% resistance to ciprofloxacin, whereas G5441, G11461 and G2992 showed 100% susceptibility to ciprofloxacin. G51 showed ciprofloxacin resistance in 70% of isolates. Similarly, tetracycline resistance was more frequently observed in genogroups G16377, G11461 and less frequently in G9184, whereas G5441, G51 and G2992 were uniformly susceptible. Ceftriaxone MICs remained consistently low across all genogroups, with only slight increases in G9184 and G16377, still well below the resistance breakpoint. Cefixime MICs also remained low, with only a slight increase observed in G16377.

## Discussion

This study presents an integrated analysis combining epidemiological, microbiological, pharmacodynamic, and molecular data on *Neisseria gonorrhoeae* infection in the Bilbao area during 2016–2017, and places these findings within the context of national and European trends.

The incidence observed in our area was high, with rates above those reported nationally and across Europe during the same period [27]. The presence of a specialized healthcare system, including dedicated STI clinics and active partner notification/contact tracing, likely contributes to a higher detection capacity and should be considered when interpreting these incidence rates.

From an epidemiological perspective, the infection mainly affected young men, with a clear predominance of MSM. This group showed a higher frequency of previous STIs, coinfections and casual sexual contacts. Our findings are consistent with recent European surveillance data and epidemiological studies reporting that MSM account for a substantial proportion of gonococcal infections and represent a key transmission group [27, 28].

Regarding clinical characteristics, most men sought care due to symptoms, whereas infections in women were mainly detected through contact tracing. This highlights the importance of partner notification, given the high proportion of asymptomatic infections among women.

Microbiological analysis showed sustained susceptibility to ceftriaxone, cefixime, and spectinomycin throughout the study period. These results are consistent with recent European surveillance data, including the Euro-GASP programme, which continues to report evolving antimicrobial resistance patterns in *Neisseria gonorrhoeae* across Europe [13, 28–32].

In contrast, high resistance rates to tetracycline and ciprofloxacin were observed, in agreement with the literature describing widespread resistance to these agents, thereby limiting their suitability for empirical therapy [13, 28–33]. In our study, ciprofloxacin resistance was significantly associated with heterosexual patients, consistent with earlier European data (2009–2016) reporting a higher prevalence of resistant strains in this group [34, 35]. However, this association should be interpreted with caution, as ciprofloxacin resistance was strongly linked to specific genogroups, particularly G9184 and G16377, which were themselves more frequently identified among heterosexual patients. Therefore, the observed association with sexual behaviour may partly reflect the distribution of resistant lineages within different sexual networks rather than an independent effect of sexual behaviour itself. Furthermore, the identification of azithromycin-resistant isolates in 2017 represented an early marker of the macrolide resistance trends that subsequently became more widespread across Europe, highlighting the value of continuous surveillance and supporting current recommendations against azithromycin-based empirical therapy [3, 36, 37].

The PK/PD analysis, performed in this study, based on the estimation of CFR values, provides additional insight into the expected efficacy of current treatments against *Neisseria gonorrhoeae*. Although PK parameters were derived from previously published models rather than local PK data, this method is commonly accepted in PK/PD analysis and allows robust estimation of treatment efficacy at the population level.

In line with this, our findings are consistent with recent PK/PD studies using Monte Carlo simulation that confirmed the excellent performance of ceftriaxone against the studied isolates, supporting its continued role as the preferred first-line treatment [16, 38]. Although cefixime also achieved favourable CFR values in our analysis, current international guidelines continue to prioritize ceftriaxone, particularly for extragenital infections [3, 4]. This recommendation is supported by evidence indicating that pharmacokinetic variability and lower antimicrobial exposure at anatomical sites such as the pharynx may reduce treatment efficacy and contribute to the higher rates of treatment failure reported in these locations [39].

Overall, these findings emphasize the importance of integrating PK/PD analysis with antimicrobial susceptibility surveillance. This approach supports evidence-based treatment recommendations and helps anticipate possible failures related to emerging resistance.

Molecular analysis revealed high genetic diversity. Several STs widely distributed nationally and internationally, such as ST5441 [40, 41], ST51 [40] and ST11461 [40, 42], were identified. In addition, 15 novel STs not previously reported were detected. Certain genogroups were associated with specific population profiles and antimicrobial susceptibility patterns. This confirms the value of molecular typing for describing the distribution of circulating lineages and their association with epidemiological and antimicrobial resistance patterns.

Some of the predominant genogroups identified in our study corresponded to lineages that have been reported across Europe. In particular, G9184 was equivalent to the internationally distributed G2400 lineage, G16377 corresponded to the internationally disseminated G1407 lineage, and G2992 represented a well-established European lineage that has been reported in multiple European countries. Notably, ST5441 and ST11461, both identified in our collection, were among the most prevalent sequence types reported in the Euro-GASP genomic survey of isolates collected in 2018 [43].

The temporal evolution of gonococcal lineages across Europe provides useful epidemiological context for interpreting our findings. In the Euro-GASP study analysing isolates collected during 2009–2010, G1407 was the dominant genogroup, accounting for approximately 23% of all isolates, and showed a significant association with MSM populations, although the lineage was also detected among heterosexual patients [26]. This genogroup was also strongly associated with decreased susceptibility or resistance to cefixime and elevated ceftriaxone MICs and was considered one of the most important multidrug-resistant gonococcal clones circulating in Europe at that time [26].

In the subsequent Euro-GASP survey based on isolates collected in 2013, G1407 remained one of the most prevalent genogroups, although its frequency had already declined significantly. Interestingly, its epidemiological profile also changed, becoming associated with heterosexual individuals and patients older than 45 years rather than MSM [44]. In contrast, G2400 emerged as a genogroup associated with MSM populations, while G2992 remained among the commonly detected lineages across several European countries [44].

More recent molecular surveillance demonstrated a continued shift in gonococcal population structure. Although G1407, G2992 and G21 were among the predominant genogroups reported in Spain during earlier surveillance periods, the predominant Spanish genogroups identified in 2018 were G14994, G9909 and G387 [45]. These findings illustrate the dynamic nature of gonococcal populations, characterized by the continuous emergence, expansion and replacement of successful lineages. In this context, the detection in our collection of G16377, corresponding to the internationally disseminated G1407 lineage, together with lineages equivalent to G2400 (represented by G9184) and G2992, indicates that the gonococcal population circulating in Bilbao during 2016–2017 shared important characteristics with the broader European population structure during a transitional epidemiological period. Furthermore, the coexistence of these lineages with ST5441 and ST11461, which later became among the most prevalent sequence types in Europe, suggests that our collection captured a phase of substantial population change [43].

In addition, Spain has previously reported internationally relevant multidrug-resistant gonococcal strains, including the ceftriaxone-resistant F89 strain belonging to ST1407, which was detected in Catalonia in 2012 and showed high-level resistance to ceftriaxone and cefixime [10]. These observations highlight the importance of maintaining integrated antimicrobial resistance and molecular surveillance systems to detect changes in circulating lineages and the emergence of resistant strains.

Under the current nomenclature system, genogroups are named after the most frequent sequence type identified in each study. As a result, the same genetic lineages may be reported under different genogroup names across studies, making direct comparisons challenging. In addition, changes in the NG-MAST database over time may further affect nomenclature consistency. Sequence types ST5119, ST4198 and ST4684 were present in the database when the molecular analyses for this study were performed but were subsequently removed following database updates implemented in 2022. Consequently, isolates belonging to this lineage are reported in the present manuscript as G2992, in accordance with the current PubMLST NG-MAST v2.0 nomenclature, to facilitate comparison with contemporary studies. This limitation should be considered when interpreting results across different settings and supports the need for more standardized or complementary molecular approaches.

This study has some limitations. First, it was conducted at a single tertiary center in northern Spain, which may limit the generalizability of the findings to other geographical areas or healthcare settings. Second, only culture-positive isolates were included, which may underestimate the true burden of infection and limit the generalizability of epidemiological findings. Third, epidemiological variables were obtained from routine clinical records, which may be subject to incomplete reporting or information bias. Fourth, molecular typing was performed using NG-MAST, a validated and widely used method, although whole-genome sequencing could provide higher discriminatory power and more detailed information on resistance determinants and transmission networks. Finally, the PK/PD analysis was based on previously published pharmacokinetic models and simulations rather than direct clinical outcome data.

Despite these limitations, this study provides robust long-term epidemiological, microbiological and molecular data contributing valuable information on the historical evolution of antimicrobial susceptibility and gonococcal population structure in our region. Although the isolate collection corresponds to 2016–2017, the dataset provides important surveillance information that helps contextualize current epidemiological trends. Current treatment decisions should continue to be guided by contemporary surveillance data and updated clinical guidelines.

In this setting, our findings have clear clinical implications, supporting the continued use of ceftriaxone as first-line therapy and reinforcing the need to avoid empirical use of agents with high resistance rates, such as ciprofloxacin.

## Supplementary Information

Below is the link to the electronic supplementary material.

Supplementary Material 1 (DOCX 24.6 KB)

## Data Availability

No datasets were generated or analysed during the current study.
