# Supplementary material for: Molecular epidemiology and pharmacokinetic–pharmacodynamic analysis of Neisseria gonorrhoeae strains in Northern Spain
Source: Eur J Clin Microbiol Infect Dis. 2026 Jul 9;45(10):3169–80. doi: 10.1007/s10096-026-05591-5 (PMC13676631; doi:10.1007/s10096-026-05591-5)
Supplement: Supplementary file 1 [file 10096_2026_5591_MOESM1_ESM.docx]

Supplementary Material

Journal: European Journal of Clinical Microbiology & Infectious Diseases

Article title: Molecular epidemiology and PK/PD analysis of *Neisseria gonorrhoeae* strains in Northern Spain

Authors:

María del Rosario Almela Ferrer^1^, María Carmen Nieto Toboso^1^˒^2^, Elisabeth Montoya Azpeitia¹, Josefina López de Munain^2,3^, Leonora Hernández Ragpa^1,2^, Íñigo Gorostiza Hormaetxe^4^, Alicia Rodríguez Gascón^5,6^, Mikel Joseba Urrutikoetxea Gutiérrez^1,2^, Andrés Canut Blasco^6,7^, Sofía Ibarra Ugarte^2,3^, José Luis Díaz de Tuesta del Arco^1,2^, Ramón Cisterna Cancer^8^, Miren Basaras Ibarzabal^8^*

^1^Department of Clinical Microbiology, Basurto University Hospital, Osakidetza Basque Health Service, Bilbao, Spain

^2^ Microbiology & Infectious Diseases Group, Biocruces Bizkaia Health Research Institute, Barakaldo, Bizkaia, Spain

^3^Department of Infectious Diseases Service, Basurto University Hospital, Osakidetza Basque Health Service, Bilbao, Spain

^4^Research Unit, Basurto University Hospital, Osakidetza Basque Health Service, Bilbao, Spain

^5^Pharmacokinetic, Nanotechnology and Gene Therapy Group (PharmaNanoGene), Faculty of Pharmacy, University of the Basque Country UPV/EHU, Spain

^6^Infectious Disease, Antimicrobial Agents, and Gene Therapy Group, Bioaraba Health Research Institute, Vitoria-Gasteiz, Spain

^7^Department of Clinical Microbiology, Araba University Hospital, Osakidetza Basque Health Service, Vitoria-Gasteiz, Spain

^8^Department of Immunology, Microbiology and Parasitology, University of the Basque Country UPV/EHU, Spain

* **Corresponding author: Miren Basaras Ibarzabal (miren.basaras@ehu.eus)**

Supplementary Table S1. Distribution of NG-MAST sequence types and corresponding *porB* and *tbpB* alleles identified in the study.

| ***porB*** | ***tbpB*** | **ST** | **n** |
| --- | --- | --- | --- |
| 30 | 18 | 5441 | 25 |
| 39 | 27 | 51 | 19 |
| 5475 | 563 | 9184 | 14 |
| 279 | 18 | 13489 | 13 |
| 6720 | 188 | 11461 | 7 |
| 90 | 953 | 5624 | 6 |
| 9518 | 110 | 16377 | 5 |
| 3104 | 29 | 5119^a^ | 4 |
| 5136 | 186 | 12547 | 4 |
| 3409 | 110 | 5619 | 4 |
| 5900 | 165 | 10420 | 4 |
| 1489 | 563 | 2400 | 3 |
| 3031 | 33 | 4995 | 3 |
| 90 | 2305 | 13467 | 3 |
| 1142 | 1531 | 8517 | 3 |
| 6748 | 953 | 11525 | 3 |
| 1808 | 29 | 2992 | 3 |
| 10681 | 18 | 18362 | 2 |
| 8285 | 18 | 14299 | 2 |
| 5942 | 563 | 10128 | 2 |
| 2577 | 29 | 4198^a^ | 2 |
| 5136 | 33 | 14994 | 2 |
| 8012 | 110 | 13863 | 2 |
| 6226 | 188 | 15724 | 2 |
| 262 | 953 | 14306 | 2 |
| 3375 | 60 | 5576 | 2 |
| 7697 | 137 | 13252 | 2 |
| 3411 | 110 | 5622 | 2 |
| 90 | 190 | 11752 | 2 |
| 1378 | 29 | 11086 | 3 |
| 5695 | 60 | 10023 | 2 |
| 7409 | 18 | 12711 | 1 |
| 19 | 563 | 15198 | 1 |
| 30 | 39 | 13858 | 1 |
| 4 | 33 | 5 | 1 |
| 435 | 188 | 645 | 1 |
| 105 | 24 | 685 | 1 |
| 2569 | 241 | 4186 | 1 |
| 3387 | 321 | 5592 | 1 |
| 2807 | 4 | 4714 | 1 |
| 90 | 29 | 758 | 1 |
| 3040 | 32 | 5118 | 1 |
| 30 | 33 | 799 | 1 |
| 174 | 27 | 239 | 1 |
| 997 | 137 | 1582 | 1 |
| 7628 | 110 | 13123 | 1 |
| 3428 | 4 | 5650 | 1 |
| 1808 | 893 | 4244 | 1 |
| 55 | 39 | 5793 | 1 |
| 8020 | 10 | 13876 | 1 |
| 3409 | 29 | 7409 | 1 |
| 8574 | 188 | 14764 | 1 |
| 279 | 29 | 7831 | 1 |
| 28 | 18 | 15680 | 1 |
| 1808 | 33 | 8435 | 1 |
| 72 | 18 | 15923 | 1 |
| 147 | 137 | 1797 | 1 |
| 3105 | 118 | 5120 | 1 |
| 2 | 16 | 2 | 1 |
| 908 | 29 | 3935 | 1 |
| 1808 | 1744 | 9654 | 1 |
| 7595 | 10 | 13070 | 1 |
| 30 | 29 | 298 | 1 |
| 7686 | 39 | 13231 | 1 |
| 182 | 4 | 1935 | 1 |
| 266 | 29 | 587 | 1 |
| 266 | 118 | 387 | 1 |
| 7954 | 563 | 13780 | 1 |
| 1489 | 27 | 10634 | 1 |
| 1873 | 39 | 4643 | 1 |
| 251 | 15 | 10932 | 1 |
| 2161 | 29 | 4684^a^ | 1 |
| 535 | 29 | 11083 | 1 |
| 8367 | 18 | 14656 | 1 |
| 1603 | 4 | 2618 | 1 |
| 90 | 18 | 621 | 1 |
| 14 | 4 | 437 | 1 |
| 2577 | 18 | 15640 | 1 |
| 1851 | 4 | 3061 | 1 |
| 9179 | 563 | 15707 | 1 |
| 6766 | 10 | 11547 | 1 |
| 1489 | 2556 | 15765 | 1 |
| 2872 | 996 | 11574 | 1 |
| 5675 | 4 | 15979 | 1 |
| 28 | 110 | 3808 | 1 |
| 10136 | 110 | 17407 | 1 |
| 1142 | 563 | 12001 | 1 |
| 279 | 39 | 19305 | 1 |
| 7249 | 110 | 12394 | 1 |
| 19 | 25 | 5268 | 1 |
| 55 | 2088 | 12415 | 1 |
|  |  | **Total** | **212** |

Note: Nineteen isolates could not be assigned an NG-MAST sequence type and were subsequently submitted to PubMLST for the assignment of new sequence types.

^a^ ST5119, ST4198 and ST4684 were valid sequence types in the NG-MAST database at the time of analysis but were removed following database updates implemented in 2022.
